# A Network Analysis-Driven Sequential Mediation Analysis of Students’ Perceived Classroom Comfort and Perceived Faculty Support on the Relationship between Teachers’ Cognitive Presence and Students’ Grit—A Holistic Learning Approach

**DOI:** 10.3390/bs13020147

**Published:** 2023-02-09

**Authors:** Tiberiu Dughi, Dana Rad, Remus Runcan, Roxana Chiș, Gabriela Vancu, Roxana Maier, Alina Costin, Gavril Rad, Sabin Chiș, Chinaza Uleanya, Macovei Crenguța Mihaela

**Affiliations:** 1 Center for Research Development and Innovation in Psychology, Faculty of Educational Sciences Psychology and Social Work, Aurel Vlaicu University of Arad, 310032 Arad, Romania; 2 Faculty of Food Engineering, Tourism and Environmental Protection, Aurel Vlaicu University of Arad, 310032 Arad, Romania; 3 Business Management, University of South Africa, Pretoria 0002, South Africa; 4 “Nicolae Bălcescu” Land Forces Academy, 550170 Sibiu, Romania

**Keywords:** holistic learning and teaching practices, community of inquiry, cognitive presence, sense of belonging, grit, network analysis, sequential mediation analysis

## Abstract

The interaction between teachers and students is critical to the learning process. Student success and learner satisfaction have consistently improved in educational situations where instructors and students connect frequently and meaningfully. The Community of Inquiry (CoI) framework, as well as the sense of belonging concept, have received a significant amount of attention from researchers investigating online learning since its debut. The current study focuses on the CoI framework in general, and in particular on studies on teaching, social, and cognitive presences in connection to students’ feeling of belonging and grit enhancement. This research investigated the opinion of 310 students at the Aurel Vlaicu University of Arad regarding their satisfaction with their teachers’ presences, their academic sense of belonging, and their grit. Our methodology followed an innovative approach. First, we employed a network analysis on all subscales’ mean scores, and then we performed a sequential mediation analysis based on both the network analysis results and the conclusions from the literature review. We tested whether students’ perceived classroom comfort and perceived faculty support sequentially mediated the relationship between teacher’s cognitive presence and students’ grit. According to the scientific literature, teacher’s cognitive presence consists of four fundamental categories: triggering events, exploration, integration, and resolution, which specifically the validation of knowledge by cooperation and reflection in a community of inquiry. We further tested if sense of belonging might mediate the relationship between teachers’ cognitive presence and students’ grit. The results show that students’ perceived classroom comfort and perceived faculty support partially and significantly sequentially mediate the relationship between teachers’ cognitive presence triggering events and students’ grit. The results are then further used to suggest possible recommendations for designing holistic learning environments in Romanian higher education institutions.

## 1. Introduction

The interaction between teachers and students is critical to the learning process. Student success and learner satisfaction have been shown to rise in educational situations when there is frequent and meaningful instructor–student interaction [1,2,3].

Holistic education incorporates a wide range of educational approaches and philosophical ideas. It strives to avoid the neglect of any key aspects of human experiences and emphasizes “wholeness” [4]. The belief that educational opportunities may aid students in developing more holistic, dynamic, and less materialistic worldviews serves as the movement’s guiding premise [5]. It also implies that educational experiences encourage a more harmonious growth of a person’s many aspects, including emotional, intellectual, spiritual, physical, social, and esthetic aspects, as well as their interactions with others, the environment, and themselves as individuals, and their exposure to a wide range of knowledge and worldviews, emotions, and reasons [6,7]. 

The holistic approach to learning and teaching is rooted in the premise that teaching represents a social interaction, in contrast to how academics could improve their education [8,9]. It is believed that a holistic learner aims for the highest degree of knowledge awareness and appreciates the benefits it provides to their life. It is believed that the goal of a holistic teacher is to empower each student with the critical faculties necessary to take appropriate action in real-world circumstances. The holistic approach to learning and teaching is defined as a social process that allows critical learners to declare ownership of the knowledge domain and its epistemology and to make knowledge claims or refutations based on that, thus permitting action in actual circumstances. A teacher’s and a student’s social interaction must consider the learner’s personal, professional, social, and human needs [10]. Along with the need to learn, these needs also include the need to be understood, to receive praise, to be a part of the learning community, and to fulfill other fundamental psychological needs [11]. 

The holistic method may be used in a variety of higher education areas, while having its beginnings in the field of computer science. Due to the holistic approach, students learn to be autonomous, self-assured, and critical. This approach aims to turn learning into a process of self-improvement by clearly recognizing oneself, the social environment of learning and teaching, and the individual needs of the learner during the encounter [9,12]. The fundamental principle of this approach is that the social context of the connection is crucial. As a consequence, it incorporates both instructors’ and students’ first-hand expertise to raise the standard of the learning environment and student achievement levels [13]. It is acknowledged that the interactions that take place during this social activity lay the groundwork for the growth of critical learners. Kelly’s Personal Construct Theory (PCT) [14] explains how the holistic approach fosters students’ development as critical thinkers in conjunction with the social exchange theory [15], which has brought theoretical insights to the study of social psychology and sociological phenomena that are crucial for comprehending trade processes at the micro- and macro-levels and the societal structures they produce.

## 2. Theoretical Framework 

With duties ranging from the selection of effective teaching strategies to the development of positive and encouraging learning environments, there is enough evidence that teachers have a key role in fostering academic achievement in students [8,16].

However, as education has moved more and more online, many of the interactional affordances that are available in a traditional classroom have either been replaced by new technology or rendered unfeasible due to time and space restrictions. For instance, face-to-face encounters have a physical refinement and immediateness that online learning lacks, since the instructor–student contact is mostly computer-mediated and typically involves asynchronous text-based exchanges. Education professionals have requested further research on the increasing responsibilities and obligations of online professors due to these substantial changes in instructor–student interactions. Recently, the idea of teaching presence has grown and gained a lot of academic interest. It is often characterized as an instructor’s virtual “visibility” in an online setting [17]. Although recent investigations on teaching presence are still in their infancy and their theories are speculative, there has been great progress in recognizing and examining the importance of creating instructional presence in online learning. [18].

Over the last few decades, a number of empirically validated recommendations have been made to organize current research on teaching and learning in order to support the best teaching strategies [19]. Recently, scientists have worked to create new models that accurately reflect the unique and cutting-edge features of online learning. Online learning scholars are intrigued by the Community of Inquiry (COI) framework. The key to understanding efficient online learning and teaching is to consider the interdependence of three types of presence: (1) cognitive presence, which refers to students’ capacity to deepen the understanding; (2) social presence, which refers to students’ capacity to show themselves as “real persons” with various traits; and (3) teaching presence, which relates to the design and facilitation of cognitive and social presences. 

The CoI (Community of Inquiry) paradigm was initially established to guide online learning research [20]. Furthermore, the CoI survey is used in a range of disciplines, such as management, education, and health care [21]. The CoI framework was developed to offer a solid theoretical foundation for both online education research and online teaching practice. It is reasonable to believe that developing a community that promotes deep learning and meaningful inquiry is required for effective online education. The relevance of this stems from the belief that young people will play key roles in the global community’s political, social, and cultural life in the future [22].

Being in accordance with established higher education norms, it provides a collaborative-constructivist method to understanding the dynamics of online learning experiences, which encourage conversation and introspection within a community of inquiry. In response to the early emphasis on social presence in computer conferencing, the CoI framework was created. The need for a comprehensive understanding of a formal online educational experience was identified at that time. The solution was to provide three highly interconnected presences: the teaching presence, the social presence, and the cognitive presence [23].

The terms “social presence” and “teaching presence” relate, respectively, to the growth, stimulation, and direction of cognitive and social functions in order to provide learning outcomes that are both intellectually and personally meaningful. The term “cognitive presence” refers to students’ ability to generate and assure meaning via persistent thinking and dialogue [24]. The impression of instructional activities to increase learning objectives and individual conceptual comprehension or results is referred to as teaching presence. The term instructional design and organization relates to how teachers view a course as they create, organize, and implement it. Its core includes everything related to the teaching and learning process, such as educational objectives, instructional methodologies, and instructional materials [25].

Researchers have discovered that promoting learning presence may also boost cognitive presence. This may indicate that, in addition to monitoring instruction, understanding development, and directing instruction, teaching presence should concentrate on developing self-regulatory skills and behaviors among online students. These results also show that CoI should promote students’ self-reliance in their education (self-regulation) [26].

Social presence is the capacity of individuals to identify with a community, converse meaningfully in a secure atmosphere, and form interpersonal relationships while maintaining their individual identities [25]. The ability to represent “real people” socially and emotionally through computer-mediated communication is referred to as social presence [27]. Cognitive presence describes a learner’s capacity to acquire a certain level of cognitive meaning through continuous communication while they are learning. Garrison describes four stages of growth in cognitive presence in online learning [28]. These stages are as follows: Students demonstrate their desire to learn more by engaging in a triggering event. This is followed by exploration, integration, and resolution, in which they investigate, integrate, and synthesize knowledge. Finally, problem solving occurs, in which students apply their knowledge to solve issues [25].

Effective online learning, according to the COI paradigm, arises from carefully planned and promoted interactions between instructional content, students, and instructors [18]. The notion of teaching presence is the most relevant to our objectives since it has substantially structured previous and current research findings into actions for effective online teachers. The COI model defines teaching presence as the planning, facilitation, and guidance of student cognitive and social processes with the purpose of producing personally meaningful and educationally desirable learning outcomes [29]. The three basic components of teaching presence are direct instructional activities, discourse facilitation, and instructional design and organization. Some refer to teaching presence as the factor that unifies a community of online learners and supports the cognitive and social connections essential for successful online learning [20,30]. When we look more thoroughly at these aspects of teaching presence, it becomes evident that more specific instructions are available for online instructors.

Prior to any interactions with students, teaching presence is established via the design and arrangement of an online course [27,31]. A teacher’s position as the principal architect and manager of learners’ educational experiences is represented in the decisions they make about the goals, scheduling, and curricular materials for a course [29]. By explicitly outlining learning objectives and maintaining a strong link between learning activities and tests, teaching professionals may successfully support students’ attempts to move through a course and take meaning from the subject being taught. Furthermore, teachers are critical in stimulating conversation between learners. Teachers play an important role in encouraging good debates by organizing class discussions, offering important issues, recognizing points of agreement, and managing student engagement [32,33]. Finally, effective and consistent use of direct instruction is required for effective teaching presence. When exercising scholarly leadership, teachers participate in direct education by presenting compelling subject matter, incorporating additional sources and points of view, and carrying out evaluative duties, such as providing feedback or assessing students’ understanding [30,34,35]. It should be noted that synchronization is not required for these teacher–student interactions; in fact, research indicates that successful asynchronous tactics in online courses typically result in greater student achievement than those that need frequent synchronous connections [36].

The COI framework’s concept of teaching presence has received a lot of support from online educators, making it the most well-known and influential model for investigating online education [37]. As a result of the COI paradigm, academics investigating online learning have produced a substantial body of academic literature [38].

Professors who have only taught in traditional classroom settings may be unfamiliar with what it takes to teach online. One of the most prominent arguments against online education is faculty members’ preference for direct connection with students and their struggle with understanding how they might impart knowledge without it. Understanding what it means to teach online can be challenging, but the Community of Inquiry approach [39] offers a framework for considering the essential elements of successful online learning. 

This study discovered a link between academic tenacity and success in school, life, and general happiness. Furthermore, academic grit outperformed general grit in terms of incremental validity in relation to these objectives. The ramifications of these results are discussed, specifically how they could drive future empirical research and practical teaching techniques [40]. According to previous research, grit is a sign of psychological well-being. This suggests that evaluating grit may be a valuable technique to identify people who are at risk of burnout or are less satisfied overall. Program administrators might use grit as a criterion to identify students who would benefit from additional assistance while attending training [41].

Since it was initially published, the Community of Inquiry (CoI) framework has drawn a lot of interest from academics studying online learning. This overview of the literature looks at both research on social, teaching, and cognitive presences and contemporary research related to the general framework. The results of this study are then used to provide some recommendations for creating holistic learning environments. According to the body of research, having teaching presence is substantially correlated with a number of crucial elements thought to promote student learning [18,42,43]. Studies examining the impact of teaching presence in online education on a regular basis show a strong correlation between students’ perceptions of learning, motivation, and satisfaction and COI teaching presence indicators, such as facilitation, course design, and direct instruction [17,44,45,46,47,48].

Additionally, it has been discovered that the presence of instructors positively affects students’ perceptions of their sense of community, and that this connection may be responsible for a considerable difference in student retention [33,49]. Additionally, studies have shown that interactions with teachers, rather than interactions with peers, are a more accurate predictor of students’ success in online learning [50,51,52]. This finding has been justified by the fact that, in order to achieve profound and significant learning gains, a strong teaching presence is necessary [30]. Conversely, in online classrooms where student engagement is expected, it might be simple for discussions to turn into drawn-out serial monologues and exchanges of personal experiences without an adequate rationale [53]. Despite the encouraging findings of research on the advantages of teaching presence, generalizations should be exercised with caution.

It is fascinating to examine recent exploratory research, particularly those examining the relationship between teaching presence and measurable learning outcomes [54,55]. Surveys of competent online students and instructors support the significance of the three elements of teaching presence even if current research can only offer preliminary advice [48,56,57]. A lot of the specific advice connected to teaching presence, such as setting students’ expectations clearly, providing frequent feedback, and giving them direct instruction that is strong, are also well supported by educational research [16]. The COI framework is used to construct and manage a variety of strategies for creating and sustaining an online teaching presence. These recommendations have been adapted from a variety of sources [17,29,58]. Some research studies indicate that the usage of e-learning platforms is significantly and favorably impacted by a sense of belonging [59].

Overall, the findings show a positive relationship between professional identity, sense of belonging, and effort persistence. For instance, the identification of an engineer directly improves learners’ sense of belonging. Both an engineering identity and a sense of belonging have a favorable direct impact on effort persistence, despite the fact that they are not statistically significant predictors of interest consistency. Additionally, a strong work ethic and an engineering identity are connected by a sense of belonging. These findings refute the notion that certain students possess grit, while others do not, and show that grit is not a singular indicator of success. If first-generation college students think they are the types of people who can study engineering and feel like they belong in the field, they are more likely to continue their efforts [60]. Academic care, belonging, and grit all show extremely strong positive correlations, suggesting a connection between these components [61]. Previous research conducted during the COVID-19 pandemic revealed that grit and belonging were connected to stress brought on by the pandemic: high stress was linked to a weaker sense of belonging and lower grit. Grit, belonging, and stress—the study’s three primary components—were all linked to institutional compassion. Institutional compassion levels were linked to a stronger sense of belonging and less stress brought on by the pandemic [62].

Having a sense of belonging in a learning community includes many different elements, including feeling like an essential part of the class’s life and activities and being welcomed, respected, and supported by instructors and students in their learning process. It demands support and respect for uniqueness and for a learner’s freedom to self-determination, and not merely a warm or favorable feeling [63]. Possessing a sense of belonging, being grateful, and maintaining an interest in learning positively influenced three measures of mental discomfort (depression, anxiety, and stress) [64]. Feeling a sense of belonging requires two main elements for students. Feelings of acceptability, necessity, and value are the first to appear. Feeling a sense of belonging also includes sentiments of association and affiliation with a team, class, or organization [65].

Students must first feel comfortable and respected by their peers and teachers in their learning surroundings before they can begin their studies. Some online learners question their ability to handle online learning because they worry about being alone in what they consider to be a bizarre online learning environment. A sense of community is in contrast to these emotions. However, according to some study on reflective individual assessments, students believe that online learning has increased their cognitive comprehensions; interpersonal skills, including collaboration; self-confidence; self-worth; connectivity; and sense of belonging. Students’ levels of self-assurance, self-efficacy, and self-esteem increase as they form solid, trustworthy relationships with their instructors and peers. According to educational research, children who feel important and valued and who actively participate in classroom life and activities have a strong feeling of belonging [66]. According to the findings of a recent study, a sense of community has a positive and significant impact on how often people use e-learning platforms [59].

## 3. Methods

### 3.1. Research Design and Methodology

The present research is an exploratory investigation intending to analyze the complex relationship between teaching presence and students’ sense of belonging and grit. 

After exploring the descriptive statistics of all research variables and establishing the gender effects on perceptions of teaching presence, sense of belonging, and students’ grit, we investigated, using a network analysis, the relationships between all research variables. The research aim of this paper is to graphically depict through network analysis the relationship between the study variables in terms of centrality metrics: betweenness, closeness, strength, and expected influence. A node’s proximity to every other node in a network is determined using the centrality of closeness. The mean of the shortest pathways linking each network node is used to compute proximity. An inverse relationship exists between a node’s centrality and the sum of its distances from every other node. The time it takes for information to flow progressively from one node to each subsequent node can be thought of as a measure of proximity. How frequently a node is on the shortest path between other nodes is measured by betweenness centrality. The measure of a node’s dependency on other nodes and the consequent capacity for control is known as betweenness centrality. Strength, which is the total of all absolute values of connections with other nodes in the network, determines a node’s impact on its close neighbors or on nodes with which it has an edge. A network is a structure that represents a set of variables and their relationships. In mathematics, it is sometimes referred to as a graph. A network is made up of nodes and edges. The nodes indicate the variables we analyze, while the edges reflect the connections between those variables. The thicker the edge between two nodes, the stronger the connection. In addition, blue lines show positive relationships, whereas red lines reflect negative relationships.

Based on the network analysis results, we tested the sequential mediation effect of students’ sense of belonging subscale related to perceived classroom comfort (SBSCC) and students’ sense of belonging subscale related to perceived faculty support (SBSPFS) on the relationship between teachers’ cognitive presence triggering event and students’ grit.

The hypothesis of this research states that perceived classroom comfort (SBSCC) and perceived faculty support (SBSPFS) represent sequential mediators in the relationship between teachers’ cognitive presence triggering event and students’ grit. We tested this hypothesis using SPSS V.26 Process Model 6, which implies the identification of a serial mediation effect, that is, a causal chain linking two mediators.

For a proper understanding of the concepts used in this research, we present in Table 1 the variables with their definitions and the abbreviations used throughout the paper.

### 3.2. Participants

In this study, participants were recruited using a convenience sampling strategy. The survey’s online version was made widely available among the Aurel Vlaicu University of Arad social media groups of current Bachelor and Master students and alumni from 9 faculties. A total of 310 valid responses were received during October and November of 2022. The respondents’ ages spanned from 19 to 61, with an average mean of 28 years. From the total of 310 responses, 20% came from male respondents and 80% came from female respondents. In terms of educational status, 67% were current Bachelor students, 21% were Master students, and 11% were alumni. All respondents gave their consent to participate in this research and agreed to aggregated data publication of their responses.

### 3.3. Instruments

The instruments used in this research are the Community of Inquiry Survey Instrument developed by [23,24], the Sense of Belonging Scale—revised, and the Short Grit Scale developed by [67,68,69]. The Community of Inquiry Survey Instrument has 34 items, corresponding to three scales: teaching presence, social presence, and cognitive presence. The teaching presence scale has three dimensions: design and organization, facilitation, and direct instruction. Al items from this scales are related to teacher activity, for example, item 1, The instructor clearly communicated important course topics; item 8, The instructor helped keep the course participants on task in a way that helped me to learn; and item 11, The instructor helped to focus discussion on relevant issues in a way that helped me to learn. The second scale, social presence, measures the following dimensions: affective expression, e.g., item 14, Getting to know other course participants gave me a sense of belonging in the course; open communication, e.g., item 18, I felt comfortable participating in the course discussions; and group cohesion, e.g., item 21, I felt that my point of view was acknowledged by other course participants. The third scale has four dimensions: triggering event, e.g., item 24, Course activities piqued my curiosity; exploration, e.g., item 26, I utilized a variety of information sources to explore problems posed in this course; integration, e.g., item 30, Learning activities helped me construct explanations/solutions; and resolution, e.g., item 33, I have developed solutions to course problems that can be applied in practice. All items were responded on 5-point Likert-type scale with 1 = strongly disagree, 2 = disagree, 3 = neutral, 4 = agree, and 5 = strongly agree.

The Sense of Belonging Scale has 4 factors, although the original scale had 5 factors (Perceived faculty support was 2 factors). The four factor are: Perceived Peer Support (8 items, e.g., If I miss class, I know students who I could get notes from); Perceived Classroom Comfort (4 items, e.g., I feel comfortable asking a question in class); Perceived Isolation (4 items, e.g., I rarely talk to other students in my class); and Perceived Faculty Support (10 items, e.g., I feel that a faculty member would be sympathetic if I was upset). The Likert-type scale has 5 points, where 1 means completely untrue, 2 means mostly untrue, 3 mans equally true and untrue, 4means mostly true, and 5 means completely true. There are neither weights or inverted scoring questions. The process of adding each item and computing the mean resulted in the creation of individual factors.

The Short Grit scale has eight items asnwered on a 5-point Likert scale: 5 = very much like me, 4 = mostly like me, 3 = somewhat like me, 2 = not much like me, and 1 = not like me at all for items 2, 4, 7, and 8, and the scores are reverse for items 1, 3, 5, and 6. For interpretation, it has to add up all the points and divide by 8. In this way it is possible to obtain a score between the maximum score 5, which means extremely gritty, and the lowest score 1, which means not at all gritty. The items are related to concentrations at work and goals. They refer to positive aspect, e.g., item 4, I am a hard worker, and item 7, I finish whatever I begin. Some of the items refer to challenges in work and concentration, e.g., items 5, I often set a goal but later choose to pursue a different one, and item 6, I have difficulty maintaining my focus on projects that take more than a few months to complete.

## 4. Results

### 4.1. Preliminary Investigation

In terms of descriptive statistics, the following means and standard deviations for the 10 subscales of the Community of Inquiry Survey were obtained: social presence group cohesion (m = 4.12, SD = 0.85), social presence affective expression (m = 4.26, SD = 0.78), social presence open communication (m = 4.26, SD = 0.80), cognitive presence exploration (m = 4.26, SD = 0.86), cognitive presence resolution (m = 4.35, SD = 0.78), teaching presence direct instruction (m = 4.39, SD = 0.86), cognitive presence triggering event (m = 4.40, SD = 0.77), cognitive presence integration (m = 4.43, SD = 0.71), teaching presence facilitation (m = 4.47, SD = 0.74), and teaching presence design and organization (m = 4.58, SD = 0.62). Thus, the highest mean was obtained for teaching presence design and organization, a concept measuring learner engagement and the structure of learning activities [70]. According to Garrison (2017), the design and organization factor includes flexibility, student accountability, and consideration of macro design elements, such as establishing clear goals and arranging activities. Establishing a secure atmosphere for students to honestly investigate and acquire information, organizing activities that will encourage a guided and balanced course of inquiry, and carefully designing examinations are other crucial components that come under this dimension. Activities related to organization, such as external e-mail, announcements, and other teacher communications that occur outside of the discussion board, are also a crucial component of this dimension [55]. Effective design and communication can direct students toward making the effort necessary for significant cognitive presence. In the last position, but still with a very high mean, is social presence affective expression, a concept reflecting the socio-emotional aspects of communication.

For the Sense of Belonging Scale—revised, the subscales obtained the following means and standard deviations: SBS perceived isolation (m = 2.57, SD = 1.00), SBS perceived faculty support (m = 3.62, SD = 1.00), SBS perceived peer support (m = 3.74, SD = 0.89), and SBS perceived classroom comfort (m = 4.03, SD = 1.01). Thus, the highest mean was obtained for SBS perceived classroom comfort. When students experience comfort in their classes, they are more inclined to relax and open up to others around them, allowing for greater creativity and critical thinking sharing. The lowest mean was obtained for SBS perceived isolation. The subjective perception of a deficit in one’s network functioning and social resources is reflected in perceived isolation. This view may include feelings of loneliness and a lack of support [71].

For the Short Grit Scale, we obtained a mean of 3.79 and a standard deviation of 0.62; this result reflects a medium persistence and enthusiasm for long-term and significant objectives in the students [72].

**Figure 1:**
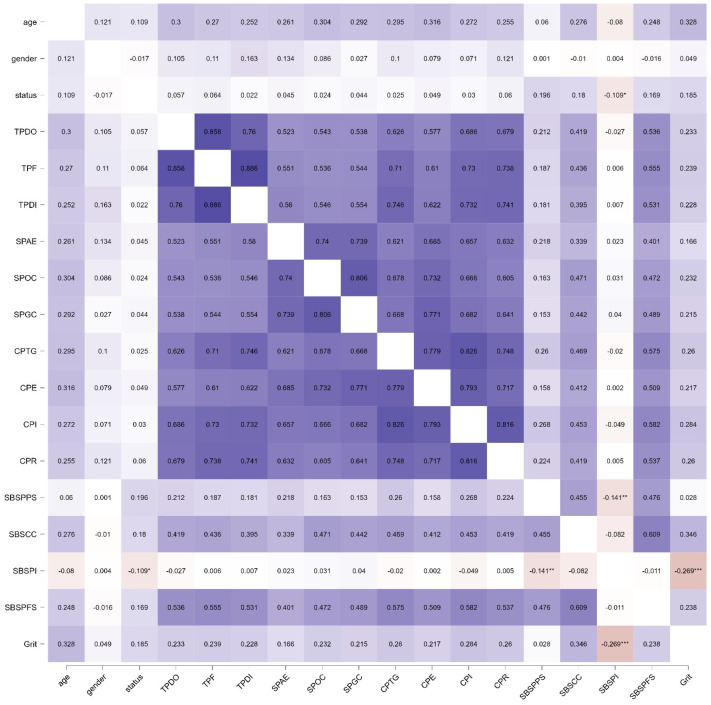
Heat map correlations. Note. * *p* < 0.05, ** *p* < 0.01, *** *p* < 0.001.

In Figure 1, we present the correlation heat map of all variables in this research. The blue color represents positive correlations and red colors represent negative correlations. The more intense the color, the more intense the correlation. There are positive associations between all variables, and the only negative and significant correlation appears to be between students’ grit and SBS perceived isolation, showing a Pearson correlation coefficient of −0.269, which is significant at *p* < 0.001. As this result reflects, the higher the students’ perceived isolation is, the lower their grit is. These results are consistent with the findings of a recent research study [73].

Due to the fact that the research sample is not gender balanced, with 80% of the respondents being females, and in order to depict the gender effect on the research variables, we employed an independent sample *t*-test, while considering the Mann–Whitney test results. The obtained results are presented in Table 2. The only difference between the male and female respondents appears to be teaching presence direct instruction, where the female respondents reported significantly higher evaluations (m = 4.46) than the male respondents (m = 4.11).

Direct instruction acknowledges the ongoing need for a knowledgeable and responsible instructor who can select the concepts and ideas that are important to learn, establish conceptual order, plan learning activities, direct the conversation, provide supplemental resources, identify misconceptions, and intervene as needed. A vital and progressive element of teaching presence in a community of inquiry is direct instruction. Students have been found to demand structure and direction [30]. Additionally, the intricacy of blended learning design options makes structure and intellectual leadership necessary. Direct instruction offers leadership that will focus conversation and resolve problems in ways that facilitation alone is not meant to achieve, given the organizational structure. Discourse that is intentional, rigorous, and fruitful is required in formal educational learning situations. Direct instruction serves this purpose. Evidence reveals that excellent leadership is substantially correlated with both satisfaction and perceived learning [38,74,75]. These activities immediately contribute to maintaining a positive social presence, which forms the basis of a community of inquiry. Teaching presence direct instruction is meant to preserve the direction and environment of education from the standpoint of social presence. Direct instruction, however, also addresses challenges with concentration and cognitive engagement. 

### 4.2. Network Analysis Results

**Figure 2:**
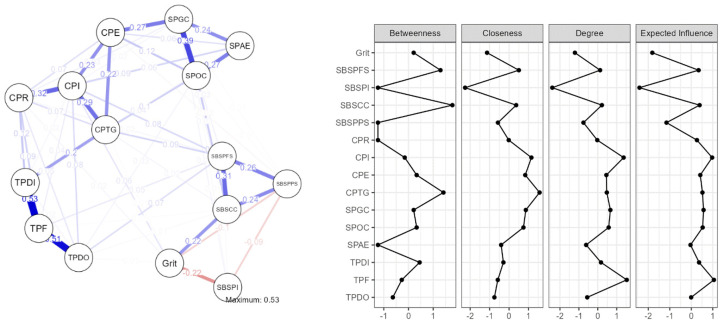
Network analysis and centrality plot.

The 15 variables were then subjected to a network analysis (Jasp software, Amsterdam, The Netherlands) to better understand their interrelationships and to analyze the complex relationship structure of the Community of Inquiry and the Sense of Belonging subscales and students’ grit (Table 3 and Figure 2).

Our network analysis involved 15 nodes, represented by all research variables: social presence group cohesion (SPGC), social presence affective expression (SPAE), social presence open communication (SPOC), cognitive presence exploration (CPE), cognitive presence resolution (CPR), teaching presence direct instruction (TPDI), cognitive presence triggering event (CPTG), cognitive presence integration (CPI), teaching presence facilitation (TPF), teaching presence design and organization, (TPDO) SBS perceived isolation (SBSPI), SBS perceived faculty support (SBSPFS), SBS perceived peer support (SBSPPS), SBS perceived classroom comfort (SBSCC), and grit, with 58 non-zero edges out of 105 and a Sparsity coefficient of 0.448.

There are four centrality metrics used to identify highly influential nodes, as shown in Figure 2 and Table 2: betweenness, closeness, strength, and expected influence [76]. In Figure 2, blue lines depict positive connections and red lines represent negative connections. The thicker the line is, the stronger the connection between nodes is.

Sense of belonging perceived classroom comfort (SBSCC) has, in terms of betweenness, the greatest influence on the flow between all variables. When activating the students’ grit network, we find the crucial importance of perceived classroom comfort and how uncomfortable classrooms interfere with students learning process.

In regard to closeness, the factor that may swiftly affect the whole network is teachers’ cognitive presence triggering event (CPTG). As students progress through the learning process, cognitive presence takes into account how they tackle new challenges, deepen their understanding, and share it with their learning community. The extent to which learners can produce and validate meaning contemplation and dialogue, known as cognitive presence, represents the most important aspect of teaching presence from the students’ perspective.

In terms of both strength and expected influence, the most influential variable over its immediate neighbors is teaching presence facilitation (TPF). Facilitating discourse refers to techniques for promoting both individual and group learning [29]. When students actively participate in group discussions with instructors and peers by personalizing, challenging, and extending the issues discussed in class, learning results are enhanced. Because of this, teachers play a crucial role in encouraging fruitful discourse by directing class discussions, posing important questions, identifying points of agreement, and regulating student involvement [33,77].

The results also indicate the significant influence of perceived isolation in the deactivation of the entire student grit network, represented by the highest negative scores obtained by perceived isolation on all centrality measures, including betweenness, closeness, strength, and expected influence.

### 4.3. Sequential Mediation Analysis Results

After performing the network analysis of the research variables, we tested the research hypothesis that perceived classroom comfort (SBSCC) and perceived faculty support (SBSPFS) represent sequential mediators in the relationship between teachers’ cognitive presence triggering event and students’ grit. We tested this hypothesis using SPSS V.26 Process Model 6, which implies the identification of a serial or sequential mediation effect.

**Figure 3:**
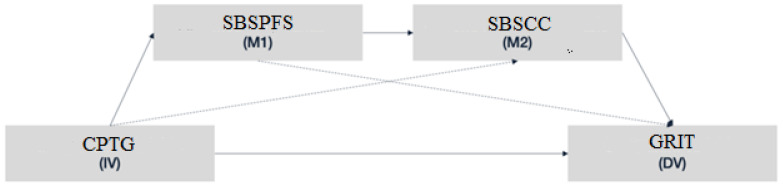
Hypothesized sequential mediation model.

In our sequential mediation model, the independent variable is teachers’ cognitive presence triggering event (CPTG), the dependent variable is students’ grit, and the two mediators are students’ sense of belonging related to perceived classroom comfort (SBSCC) and students’ sense of belonging related to perceived faculty support (SBSPFS). The two mediators increasingly enhance the prediction of the dependent variable, students’ grit level. The schematic below (Figure 3) represents the sequential mediation model of the present study, which has been designed based on theory and previous findings.

The results indicate a significant indirect effect of CPTG on students’ grit through the mediators, students’ sense of belonging related to perceived classroom comfort (SBSCC) and students’ sense of belonging related to perceived faculty support (SBSPFS) (b = 0.069, t = 3.399), supporting our hypothesis. Furthermore, the direct effect of CPTG on students’ grit in the presence of the mediators is also significant (b= 0.1061, t = 1.9955, *p* < 0.05). Hence, there is partial sequential mediation of students’ sense of belonging related to perceived classroom comfort (SBSCC) and students’ sense of belonging related to perceived faculty support (SBSPFS) on the relationship between CPTG and student’s grit. The results of the mediation model are presented in Table 4.

The sequential mediation model explains an overall 13% of the variance in students’ grit, representing a very high percentage. This result is supported by Community of Inquiry framework and by practical results reported in the scientific literature in reference to the relationship between students’ sense of belonging and grit.

In conclusion, the two mediators partially mediate the relationship between CPTG and students’ grit (IE = 0.069, 95% CI: LL = 0.0321 to UL = 0.1123), indicating that students with a high perception of teachers’ cognitive presence triggering event, as well as highly perceived faculty support and perceived classroom comfort, are more likely to possess greater grit.

## 5. Discussion and Implications

By looking at the educational process through the lenses of the Community of Inquiry framework and sense of belonging, this research aimed to identify models that have a significant indirect effect on students’ grit. The results show that perceived classroom comfort (SBSCC) and perceived faculty support (SBSPFS) represent sequential mediators in the relationship between teachers’ cognitive presence triggering event and students’ grit. These results are highly supported by other research results and by the theoretical framework of the Community of Inquiry.

For example, in the research by Stenbom (2018) [78], the two basic concepts of community and inquiry build a realistic organizational framework of enduring ideas and practices, with the aim of directing online educational activity [79,80,81,82].

In their seminal work on teaching presence in 2001, Anderson, Rourke, Garrison, and Archer [29] compared teaching in an online classroom to a one-room schoolhouse. This comparison demonstrates that an online teacher, as opposed to being a passive observer, plays a substantial and varied role. Planning, supporting, and managing social and cognitive processes, with the intention of achieving learning results that are both personally engaging and educationally worthwhile, is what is meant by “teaching presence” [29]. Each of these three functions has a variety of related activities. Experienced online educators are aware that upfront course design is more crucial for online learning than it is in many traditional classroom settings. The course design process includes locating and developing curricular resources, organizing lesson plans, defining assignment guidelines and assessment criteria, and other activities. The best courses provide these elements in a way that is obvious and supports what [29] refers to as the course’s grand design. This idea may be referred to as course architecture in the context of systems theory.

Facilitation involves frequently monitoring and commenting on the posts and work of students to keep them engaged, motivated, and interested in the course materials. Helping students comprehend the types of contributions they should be making depends on an instructor’s involvement in this aspect.

A teacher leads intellectually and academically by directing the cognitive process [29]. Assuming a more consultative position does not mean that academics should cease using their in-depth topic knowledge to assess students’ comprehension, assist them by offering resources, and help in removing their misunderstandings. The teaching presence concept’s fundamental assumption is that instructors are crucial to online students’ learning, both in terms of the initial creation of integrated learning experiences and in the ongoing support of learning mechanisms based on interaction. In online learning, there is a distinction between quantity and quality of communication, ensuring that the messages truly contribute to a meaningful debate or guiding thought processes in the classroom.

Campus-based learning places a high importance on sense of belonging, and research shows that it is closely related to higher student achievement, greater learner satisfaction, and lower attrition rates [66]. Additionally, there are some links between students’ sense of community in the classroom and their task difficulty, internal motivation, and academic self-efficacy. Additionally, there are links between students’ perceptions of belonging in a class and teachers’ friendliness and approachability, their degree of organization, and how highly they respect student engagement [83].

Researchers have discovered that being a part of a school positively and significantly affects a variety of motivating factors in learning environments, such as expectations for success, recognition of academic achievement, and self-reported effort [63]. Increasing student performance and school engagement may both be improved by increasing school belonging [84]. Therefore, educational programs, practices, and research must take this into consideration. For students to succeed academically and to be engaged, their sense of belonging at school must be improved [85].

Some explanations claim that grit is a higher-order personality characteristic that is distinct from other qualities, such as conscientiousness, and that is highly predictive of performance and success. Grit is linked to adaptive outcomes in both children and adults, including general self-efficacy, work satisfaction, career retention, and positive affect, according to Credé et al. (2017) [72]. Passion and persistence for long-term goals are two characteristics of grit. It was proposed to provide an explanation for why certain people are adamant on realizing their potential. According to research findings, accomplishing challenging goals requires both skills and the deliberate, sustained use of talent over time [67,85]. The hierarchical dispositional feature known as grit is made up of two basic elements: persistence of effort and consistency of interest. Effort persistence entails overcoming challenges in order to accomplish difficult goals, whereas interest consistency emphasizes keeping a constant focus on goals across time [68,69]. The tenacity of the effort component of grit has been proven to be more important in a collectivist society than consistency of interest [86]. Since it is a predictor of success and academic performance, personality psychologists have focused a lot of attention on grit, or the drive and tenacity for long-term goals. Grit is sometimes criticized for being merely another measure of self-control or diligence [87].

According to Sustainable Development Goal 4, all governments worldwide need to strive to ensure that all children have access to high-quality education at all levels by 2030 [88]. To genuinely highlight children’s rights is the first step. The following actions are required to meet the SDG4 targets: developing inclusive educational policies with genuine community support from all stakeholders; fostering an inclusive culture with a thorough understanding of child development; investing in teacher preparation; and instilling a strong belief in and positive attitudes toward equity in educational services [88]. All pertinent educational components must be ready for holistic education to move from a concept to a reality [89]. Based on prior experiences, research findings, and international best practices, it suggests that cooperation is required as a component of the process in order to guarantee that all students obtain a comprehensive education. In addition to assisting and overseeing instructors, this process includes putting the required programs, tactics, and evaluations in place.

## 6. Conclusions and Limitations

Overall, this is the first mediation study that looks at the students’ sense of belonging subscale related to perceived classroom comfort (SBSCC) and the students’ sense of belonging subscale related to perceived faculty support (SBSPFS) as sequential mediators in the relationship between teachers’ cognitive presence triggering event and students’ grit.

Significant evidence demonstrates that positive instructor–student connections are associated with higher student engagement and success. Although this has not been a focus of the literature, we hypothesize similar benefits for instructors. Our hypothesis, which asserts that individuals increase effort and performance in work contexts when they have positive interactions, is supported by both self-determination theory and leader–member exchange theory.

According to a study conducted in the United States [77], when a teacher shows students that they are progressing and demonstrates positive attitudes toward them, the students are more likely to make further efforts and, as a result, are more likely to complete more difficult tasks. Earlier research has shown that when students feel connected to their teachers and environment, they are more driven to study. However, the reverse relationship is also verified, namely that when teachers and students get along well, they use higher-quality teaching techniques. The most effective teachers are those who show an interest in getting to know their students better, who are approachable, and who show that they enjoy what they do. Thus, the emotional capital created in the classroom is changed into academic capital. Students take on more difficult academic tasks, behave in a way that is suitable for the classroom, such as seeing their teachers, and go beyond their teachers’ expectations when they perceive that their teachers care about them [77].

## Figures and Tables

**Table 1:** Variables used in the research.

Nr. Crt.	Variable	Definition	Abbreviation
1.	Teaching presence design and organization	decisions on course objectives, schedules, and curricular materials	TPDO
2.	Teaching presence facilitation	encouraging productive discourse by directing class discussions, posing important questions, identifying areas of agreement, and controlling student involvement	TPF
3.	Teaching presence direct instruction	practicing intellectual leadership, such as offering feedback or measuring student knowledge, and undertaking evaluative activities, such as providing feedback or assessing student understanding	TPDI
4.	Social presence affective expression	students convey their personal expressions and beliefs	SPAE
5.	Social presence open communication	learners gain mutual awareness and recognition skills	SPOC
6.	Social presence group cohesion	students develop and maintain a sense of group devotion	SPGC
7.	Cognitive presence triggering event	a topic or challenge is provided, preferably one that would pique the interest of students	CPTG
8.	Cognitive presence exploration	students strive to comprehend the nature of one particular aspect	CPE
9.	Cognitive presence integration	the information obtained during the exploration phase becomes consolidated	CPI
10.	Cognitive presence resolution	based on the work completed during the integration phase, a solution is selected	CPR
11.	Perceived faculty support	students’ perceived social support from teachers on campus	SBSPFS
12.	Perceived peer support	peer support is an umbrella terminology for a variety of approaches and techniques, such as peer tutoring, coaching, listening, mentoring and counseling	SPSPPS
13.	Perceived classroom comfort	students experience comfort in their classes	SBSCC
14.	Perceived isolation	reflects one’s subjective impression of a lack of network functioning and social resources	SBSPI
15.	Grit	working tirelessly on tasks and retaining effort and interest over years despite failure or difficulty	Grit

**Table 2:** Gender effect.

Independent Sample *t*-Test							
						95% CI for Effect Size	
	Test	Statistic	df	*p*	Effect Size	Lower	Upper
TPDO	Student	−1.854	308.000	0.065	−0.260	−0.536	0.016
	Welch	−1.542	81.106	0.127	−0.235	−0.512	0.043
	Mann–Whitney	7357.000		0.375	−0.065	−0.221	0.093
TPF	Student	−1.947	308.000	0.052	−0.273	−0.549	0.003
	Welch	−1.725	85.841	0.088	−0.256	−0.533	0.022
	Mann–Whitney	6922.000		0.112	−0.121	−0.273	0.038
TPDI	Student	−2.892	308.000	0.004	−0.406	−0.682	−0.129
	Welch	−2.447	82.302	0.017	−0.371	−0.650	−0.089
	Mann–Whitney	6443.000		0.016	−0.182	−0.330	−0.025
SPAE	Student	−2.375	308.000	0.018	−0.333	−0.609	−0.057
	Welch	−2.003	82.048	0.048	−0.304	−0.582	−0.024
	Mann–Whitney	6784.000		0.080	−0.138	−0.290	0.020
SPOC	Student	−1.510	308.000	0.132	−0.212	−0.487	0.064
	Welch	−1.413	90.828	0.161	−0.205	−0.481	0.072
	Mann–Whitney	7026.500		0.172	−0.107	−0.261	0.051
SPGC	Student	−0.478	308.000	0.633	−0.067	−0.342	0.208
	Welch	−0.473	97.225	0.637	−0.067	−0.342	0.209
	Mann–Whitney	7555.000		0.613	−0.040	−0.197	0.118
CPTG	Student	−1.766	308.000	0.078	−0.248	−0.523	0.028
	Welch	−1.638	89.985	0.105	−0.238	−0.515	0.040
	Mann–Whitney	6745.500		0.063	−0.143	−0.294	0.015
CPE	Student	−1.392	308.000	0.165	−0.195	−0.471	0.080
	Welch	−1.386	97.832	0.169	−0.195	−0.471	0.082
	Mann–Whitney	6776.000		0.076	−0.139	−0.291	0.019
CPI	Student	−1.254	308.000	0.211	−0.176	−0.451	0.100
	Welch	−1.204	93.563	0.232	−0.172	−0.448	0.104
	Mann–Whitney	7099.500		0.197	−0.098	−0.252	0.061
CPR	Student	−2.148	308.000	0.033	−0.301	−0.577	−0.025
	Welch	−1.941	87.555	0.056	−0.286	−0.563	−0.007
	Mann–Whitney	6781.500		0.073	−0.139	−0.290	0.020
SBSPPS	Student	−0.021	308.000	0.983	−0.003	−0.278	0.272
	Welch	−0.021	100.915	0.983	−0.003	−0.278	0.272
	Mann–Whitney	7721.000		0.813	−0.019	−0.176	0.139
SBSCC	Student	0.182	308.000	0.855	0.026	−0.249	0.301
	Welch	0.197	109.245	0.845	0.027	−0.249	0.302
	Mann–Whitney	7759.500		0.857	−0.014	−0.172	0.144
SBSPI	Student	−0.069	308.000	0.945	−0.010	−0.285	0.265
	Welch	−0.067	94.371	0.947	−0.010	−0.285	0.265
	Mann–Whitney	7847.000		0.969	−0.003	−0.161	0.155
SBSPFS	Student	0.275	308.000	0.784	0.039	−0.237	0.314
	Welch	0.282	101.939	0.778	0.039	−0.236	0.314
	Mann–Whitney	8000.000		0.842	0.016	−0.142	0.174
Grit	Student	−0.854	308.000	0.394	−0.120	−0.395	0.155
	Welch	−0.864	99.852	0.389	−0.121	−0.396	0.155
	Mann–Whitney	7349.500		0.413	−0.066	−0.222	0.092

Note. For the Student’s *t*-test and Welch’s *t*-test, effect size is given by Cohen’s d. For the Mann–Whitney test, effect size is given by the rank biserial correlation.

**Table 3:** Network analysis centrality measures.

Centrality Measures per Variable				
	Network			
Variable	Betweenness	Closeness	Strength	Expected Influence
TPDO	−0.636	−0.755	−0.559	−0.006
TPF	−0.274	−0.579	1.530	1.054
TPDI	0.451	−0.292	0.169	0.363
SPAE	−1.239	−0.408	−0.615	−0.035
SPOC	0.330	0.739	0.574	0.529
SPGC	0.209	0.860	0.664	0.572
CPTG	1.416	1.567	0.477	0.519
CPE	0.330	0.831	0.453	0.419
CPI	−0.153	1.147	1.366	0.971
CPR	−1.239	−0.016	−0.029	0.263
SBSPPS	−1.239	−0.571	−0.757	−1.153
SBSCC	1.778	0.365	0.217	0.388
SBSPI	−1.239	−2.259	−2.403	−2.410
SBSPFS	1.296	0.502	0.123	0.340
Grit	0.209	−1.132	−1.210	−1.813

**Table 4:** Sequential mediation summary.

Total Effect (CPTG→Grit)	Direct Effect (CPTG→Grit)	Relationship	Indirect Effect	Confidence Intervals		*t*	Conclusion
				Lower Bound	Upper Bound		
0.2088 *p* < 0.0001	0.1061 *p* < 0.0469	H1: CPTG → SBSPFS → SBSCC → Grit	0.069	0.0321	0.1123	3.399	Partial mediation

## Data Availability

The authors will make the raw data supporting the conclusions of this study available without restriction.
